# Prenatal Harmattan exposure and birth size: identifying sensitive windows in pregnancy

**DOI:** 10.3389/fgwh.2026.1576529

**Published:** 2026-05-18

**Authors:** Gabriella Y. Meltzer, Seyram Kaali, Kenneth A. Ae-Ngibise, Mohammed Mujtaba Nuhu, Anna M. Modest, Ellen Boamah, Steven Chillrud, Oscar Agyei, Patrick L. Kinney, Abena Konadu Yawson, Darby Jack, Louisa Iddrisu, Jones Opoku-Mensah, Alison G. Lee, Blair J. Wylie, Kwaku Poku Asante

**Affiliations:** 1 Department of Obstetrics and Gynecology, Columbia University Irving Medical Center, New York, NY, United States; 2 Department of Environmental Science, American University, Washington, DC, United States; 3 Department of Health Studies, American University, Washington, DC, United States; 4 Kintampo Health Research Centre, Research and Development Division, Ghana Health Service, Kintampo, North Municipality, Ghana; 5 Department of Obstetrics and Gynecology, Beth Israel Deaconess Medical Center, Brookline, MA, United States; 6 The Earth Institute, Columbia University, New York, NY, United States; 7 Department of Environmental Health, Boston University School of Public Health, Boston, MA, United States; 8 Department of Environmental Health Sciences, Columbia University Mailman School of Public Health, New York, NY, United States; 9 Division of Pulmonary, Critical Care and Sleep Medicine, Icahn School of Medicine at Mount Sinai, New York, NY, United States

**Keywords:** birth length, birth weight, Ghana, Harmattan, head circumference, PM_2.5_, pregnancy

## Abstract

**Introduction:**

The Harmattan season (approximately December to March) in Western Africa is characterized by dry, dusty trade winds blowing from the Sahara Desert toward the Gulf of Guinea and is associated with marked increases in fine particulate matter and other air pollutants, cooler temperatures, and crop reductions. The season has been linked with several negative health effects, though impacts on pregnancy outcomes are unknown. We leveraged data from the Ghana Randomized Air Pollution and Health Study (GRAPHS) to determine whether prenatal exposure to the Harmattan season impacts newborn size and whether there are critical windows of exposure.

**Methods:**

GRAPHS enrolled 1,414 pregnant women from Kintampo, Ghana from 2013 to 2015. We employed distributed lag models (DLMs) to examine time-varying associations between prenatal exposure to the Harmattan season (yes/no) for each week of gestation and birth weight, length, and head circumference among infants liveborn ≥ 37 weeks.

**Results:**

Analyses included *n* = 1,261 mother-infant pairs. Harmattan exposure was associated with smaller head circumference across gestation and DLMs identified sensitive windows in weeks 1–11 and 15–34. These effects were only significant among infant males. No impact of Harmattan on birth weight or birth length was identified.

**Discussion:**

Our analyses suggest prenatal exposure to the Harmattan season is associated with negative impacts on newborn size and specifically head circumference; exposure during certain gestational windows appears to have a greater impact than others. Climate change threatens to make Harmattan more severe secondary to increased desertification; we therefore need a better understanding of its health effects during pregnancy.

## 1 Introduction

The Harmattan season in West Africa is the result of dry, dusty trade winds blowing from the northeast of the Sahara Desert towards the Gulf of Guinea and occurs from around December through March each year. Air pollution is worse during Harmattan compared with other times of the year, as demonstrated by measured increases in ambient particulate matter concentrations in several studies from West Africa (1–3). In one study conducted in the capital city of Togo, average fine particulate matter (PM_2.5_) concentrations increased above baseline by 21% to 58% and exceeded the World Health Organization's recommended guidelines for daily PM_2.5_ exposure almost every day during the Harmattan season (2). Our own group has documented seasonal variation in daily personal exposure measurements to carbon monoxide and PM_2.5_ among a cohort of pregnant women, with peaks in the months of December to March (4). In addition to ushering in air pollutants, this dry season also brings cooler temperatures during both the day and night, as well as lower humidity (5). Fire is widely used during the Harmattan to clear land of brush and agricultural residues, contributing to ambient air pollution (6); finally, home fires are used by the population for warmth, further contributing to air pollution within households (7). The season is also characterized by an increased risk of crop failures and food insecurity (8, 9).

Adverse health consequences have been associated with the Harmattan season, including exacerbation of pre-existing allergies, asthma, hypertension, and congestive heart failure, as well as a rise in infant and child mortality, strokes, epistaxis requiring hospitalization (from lower humidity), postnatal depression, and burns (9–15). Both viral and bacterial airborne infections are also reportedly more frequent during Harmattan winds, with documented increases in influenza (16), pneumococcal disease (17), COVID-19 (5), and meningococcal meningitis (18–20). Saharan dust can be carried by the Harmattan winds beyond Northwestern Africa to Europe and North and South America, and researchers in Spain reported that the increase in coarse particulate matter exposure (PM_10–2.5_) increased daily mortality in the entire population by 8.4% (95% confidence interval 1.5%–15.8%) on Harmattan days compared to 1.4% (95% CI −0.8%–3.4%) on non-Harmattan days (21).

There is an extensive and growing literature on the negative effects of exposure to ambient air pollution during pregnancy, including work from our own group in Ghana (22–24). However, specific evaluations of the seasonal effects of Harmattan on pregnancy health are sparse, even in West Africa. A study from a single tertiary care hospital in northwest Nigeria evaluating the epidemiology of peripartum cardiomyopathy reports peaks in hospital admissions for peripartum cardiomyopathy during both the rainy season and the Harmattan season (25). Outside of Africa, researchers from Spain have evaluated whether Saharan dust episodes affect birth outcomes with conflicting results. One group from Spain noted no effect of the number of Saharan dust cloud days during pregnancy (entire pregnancy and trimester-specific) on pregnancy complications, including preeclampsia, bacteriuria, or birth weight (26). Other researchers from Spain report that days with Saharan dust intrusion increased measured levels of PM_10_, NO_2,_ and O_3_ and were associated with increased acute risk for preterm birth and low birth weight (27). In the Caribbean nation of Guadalupe, PM_10_ concentrations increased with Saharan dust events as documented by a monitoring station in the capital city; in turn, the proportion of intense dust events during pregnancy was associated with increased odds of preterm birth (28).

**Figure 1:**
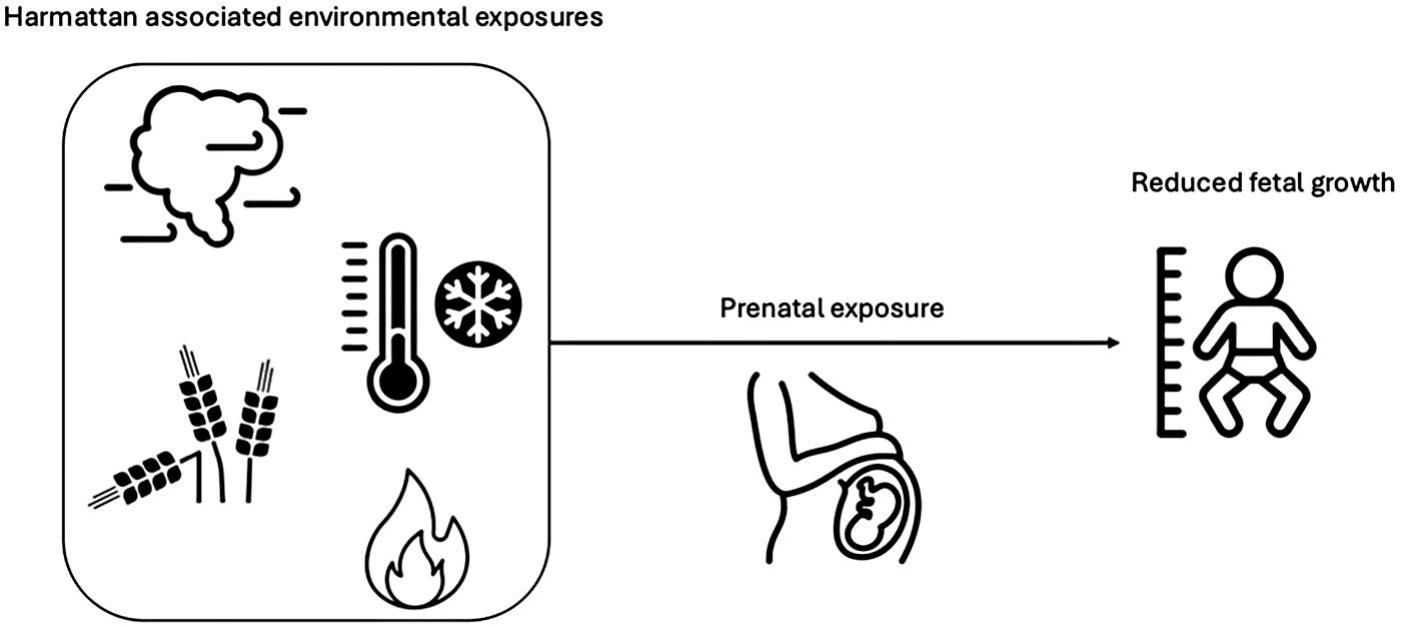
Conceptual framework linking Harmattan exposure to fetal growth.

Given the need for greater research on the effects of Harmattan on pregnancy health in West Africa, we utilize data from the Ghana Randomized Air Pollution and Health Study (GRAPHS, NCT01335490) to conduct an exploratory evaluation of the impact of the Harmattan season on infant size at birth, specifically birth weight, length, and head circumference. We leverage the temporality of the Harmattan to identify whether there may be specific windows of vulnerability during pregnancy to this season of increased air pollution, lower temperatures and humidity, and associated crop reductions and food insecurity (Figure 1).

## 2 Methods

Included in this analysis are participants from GRAPHS, a cluster-randomized controlled trial of clean cookstove interventions introduced during pregnancy that was conducted between August 2013 and March 2016 in the Kintampo North Municipality and Kintampo South District of the Brong Ahafo Region of Ghana, details of which have been published previously (4, 29). Briefly, pregnant women residing in study communities were eligible for study inclusion if they were the primary household cook, a non-smoker, and pregnant with a live singleton fetus at gestational age ≤24 weeks as determined by ultrasound (30). Procedures were approved by human studies and institutional ethics committees at Kintampo Health Research Centre (KHRC, IRB0004854), the Columbia University Mailman School of Public Health (IRB AAAR4373), and the Icahn School of Medicine at Mount Sinai (IRB HSM-14-00572), and written consent was obtained from all pregnant women.

Following a baseline exposure assessment, *n* = 1,414 pregnant women were randomized to either a liquefied petroleum gas or improved biomass cookstove arm or continued cooking on a traditional, open-fire cookstove. Birth weight was recorded within 24 h at the place of delivery by community-based field workers and measured to the nearest 0.01 kg (Tanita digital scale model BD-590, Tanita, Illinois, USA). Any birth weight not measured within 24 h of birth was considered missing. Birth length was measured using the Ayrton Infantometer Model M-200 (Ayrton, Minnesota, USA) and head circumference was measured using a paper tape (“lasso”) from the Child Growth Foundation in London. Both were measured and recorded to the nearest 0.1 cm (23). In the primary intention-to-treat results, neither birth weight nor other birth anthropometrics were improved by the cookstove interventions (23).

### 2.1 Harmattan season analysis

**Figure 2:**
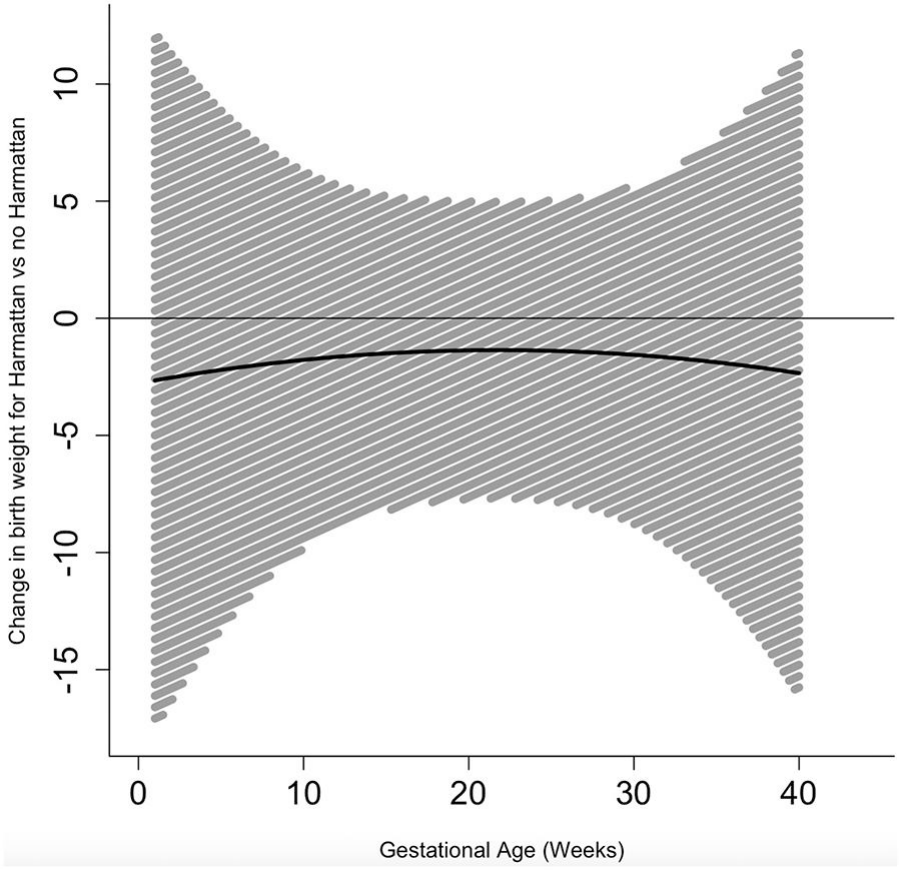
Time-varying associations between Harmattan and birth weight (in grams).

The Harmattan season was approximated and defined as the four months of December, January, February, and March. This was consistent with findings from the original GRAPHS trial that documented seasonal trends in elevated levels of individual maternal PM_2.5_ with periodicity corresponding to the Harmattan season from November 2013 to February 2016 (4), (Figure 2B). Each week of pregnancy, based on ultrasound gestational dating, was assigned as either occurring during the Harmattan or not occurring during the Harmattan. We employed an extension of the distributed lag model (DLM) to evaluate sensitive windows for the effect of prenatal Harmattan exposure on birth weight, birth length, and head circumference, considered separately (31, 32). DLMs allow estimation of time-varying effects of an exposure while adjusting for exposure at other time points, as well as relevant covariates. They have been applied previously in environmental health research, including by our group (33–35). In this analysis, the Harmattan season was treated as a dichotomous exposure variable. DLMs require exposures to be measured at fixed time points to fit a smoothed function. Our fixed interval was therefore one week. Since DLMs do not allow for missing data, once a delivery occurred, the seasonal attribution (Harmattan yes/no) at the time of the birth was carried forward through 41 weeks’ gestation. We restricted analyses to live singleton births ≥ 37 weeks’ gestation. The decision to limit to term birth was made for two reasons. First, the DLM model is unable to handle missing data. Second, preterm birth may mediate the association between Harmattan-related environmental exposures, such as elevated ambient particulate matter, and reduced anthropometrics (28). DLM is also unable to handle mediation. This restriction to term birth reduced inter-individual variability in gestational length.

The association of Harmattan exposure on the considered outcome (e.g., birth weight) for each given week in gestation was plotted along with 95% confidence intervals. A sensitive window was identified if the effect estimates and confidence intervals did not cross zero. Models were adjusted for Harmattan exposure at all other weeks of gestation, as well as maternal age, parity at enrollment, infant sex, maternal body mass index (BMI) at enrollment, self-reported ethnicity (A, B, C, D), asset index [a measure of relative socioeconomic status (36)], the number of antenatal visits (≥4 vs. <4), and placental malaria as determined by histopathology. These covariates were chosen as relevant determinants of birth weight in this population, potential confounders to the Harmattan-birth size relationship, and in line with our prior exposure-response analyses in GRAPHS (24). We did not adjust for measured personal exposure to fine particulate matter, which was measured once during pregnancy throughout the GRAPHS trial, as we hypothesized that elevated levels of ambient particulate matter during the Harmattan season lie on the causal pathway between Harmattan exposure and birth size. Adjusting for fine particulate matter may partially mediate the impact of Harmattan exposure on birth size and therefore attenuate its effect. We tested DLMs with varying degrees of freedom (df = 2, 3, 4, and 5) to determine the optimal model structure and tested model fit using AIC. We then estimated the cumulative effects of Harmattan season exposure, which allowed us to aggregate the time-varying impacts across pregnancy to estimate how continuous Harmattan exposure throughout the entire gestational period would impact birth size. This cumulative effect was determined by summing the time-specific coefficients from the DLM across all gestational weeks, reflecting an extrapolated cumulative DLM effect as opposed to a directly observed contrast. We also stratified all the models that were significant in the full cohort by infant sex and placental malaria based on our prior work. We observed sex-specific effects in previous GRAPHS trial analyses linking household air pollution to maternal and child health outcomes, including child lung function and pneumonia (37), infant growth trajectories (38), postpartum blood pressure (39), and child mtDNA content and leukocyte telomere length (40). Analyses were conducted using R Statistical Software (v4.2.2.; R Core Team 2022).

## 3 Results

As previously reported, GRAPHS enrolled 1,414 pregnant women from August 2013 to August 2015, and data collection concluded in March 2016, spanning 3 Harmattan seasons. The cohort resulted in 1,306 live births beyond 28 weeks’ gestation, of which 1,261 were term (≥ 37 weeks’ gestation) live births used for this analysis (Supplementary Figure S1). On average, participants were 28 years old, had previously given birth 3 times, and were enrolled in the study at 16 weeks’ gestation. Most participants identified with ethnic group C (65%), had completed primary education (59%), were married (56%), and had attended at least 4 antenatal visits (71%). Nearly a quarter had been diagnosed with placental malaria (24%). Among those who delivered at term, the average gestational age at delivery was 39.4 weeks (SD 1.3) and the average birth weight was 2,920 grams (SD 440.5). Average birth length and head circumference were 46.7 (SD 3.6) and 33.6 (SD 2.5) centimeters, respectively. Characteristics and birth outcomes of the included cohort are outlined in Table 1.

**Table 1:** Baseline characteristics and birth outcomes among GRAPHS participants who delivered at ≥ 37 gestational weeks, *N* = 1,261.

Characteristic	n (%); mean (SD), range
A. Baseline characteristics	
Age	28.1 (7.2), 14.7–50.2
BMI (kg/m^3^)	23.3 (3.2), 14.1–38.5
Parity	2.8 (2.2), 0–13
Asset index	0 (2.0), −3.0–13.1
Gestational age at enrollment (weeks)	15.8 (4.3), 6–26
Ethnicity	
A	218 (17.3%)
B	167 (13.2%)
C	820 (65.0%)
D	56 (4.4%)
Smoker in household or compound	265 (21.0%)
Diagnosis of placental malaria	272 (24.0%)
Antenatal care visits	
<4	361 (28.8%)
≥4	892 (71.2%)
Completed primary education	746 (59.2%)
Married	710 (56.3%)
B. Birth Outcomes	
Infant Sex	
Female	623 (49.4%)
Male	638 (50.6%)
Gestational age at delivery (weeks)	39.4 (1.3), 37–44
Birthweight (grams)	2,920 (440.5), 1,500–4,500
Birth Length (cm)	46.7 (3.6), 31.0–64.0
Head Circumference (cm)	33.6 (2.5), 21.5–45

### 3.1 Time-varying effects of Harmattan on birth size

**Figure 3:**
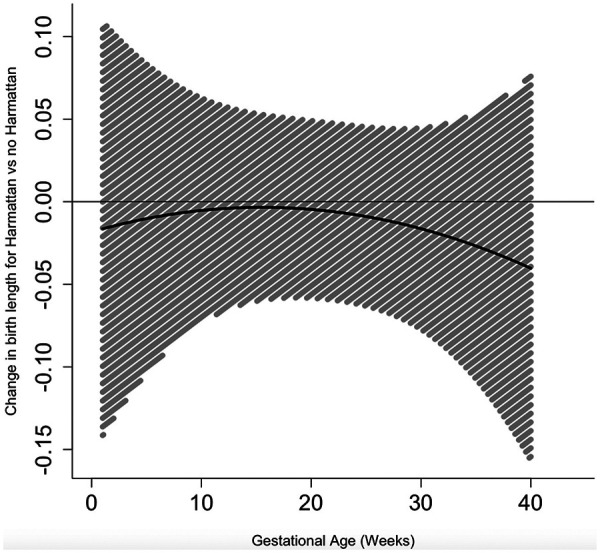
Time-varying associations between Harmattan and birth length (in centimeters).

**Figure 4:**
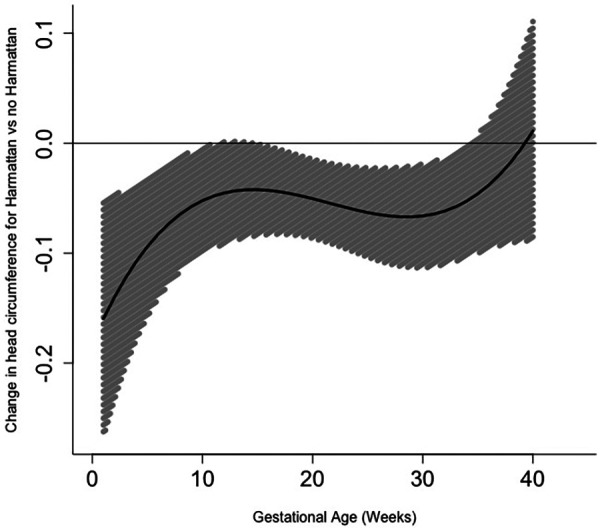
Time-varying associations between Harmattan and head circumference (in centimeters).

Exposure to the Harmattan season during pregnancy did not have time-varying effects on birth weight (Figure 2, Supplementary Table S1) or birth length (Figure 3, Supplementary Table S2) at any point across gestation. However, exposure to the Harmattan season during gestational weeks 1–11 and 15–34 had significant negative effects on infant head circumference (Figure 4, Supplementary Table S3). Models stratified by infant sex showed that exposure to Harmattan only had significant effects on head circumference among male infants.

### 3.2 Overall effects of Harmattan across gestation

In addition to determining windows of vulnerability across gestation, we also examined what the potential effect of Harmattan would have been on newborn size had women been exposed for the entirety of pregnancy, adjusting for the aforementioned confounders. Hypothetical exposure to Harmattan during all weeks of gestation compared to no Harmattan exposure was not associated with birth weight (ß −70.56 g; 95% CI −385.29 g, 244.18 g) or birth length (ß −0.53 cm; 95% CI −3.18 g, 2.13 g). It was, however, associated with an average 2.39 cm smaller head circumference (95% CI −4.17 cm, −0.61 cm; *p* = 0.009), corresponding to approximately −0.96 standard deviations based on the sample distribution. This association was only significant among those giving birth to males (ß −2.67 cm; 95% CI −5.24 cm, −0.09 cm; *p* = 0.04), equivalent to approximately −1.06 standard deviations based on the male-specific sample distribution (Table 2).

**Table 2:** Overall cumulative effects of harmattan on birth anthropometrics based on distributed lag models, *N* = 1,261.

Birth anthropometric	ß (95% confidence interval)^a^
Birth weight (g)	−70.56 (−385.29, 244.18)
Birth length (cm)	−0.53 (−3.18, 2.13)
Head circumference (cm)	−2.39 (−4.17, −0.61)**
Males	−2.67 (−5.24, −0.09)*
Females	−1.98 (−4.46, 0.50)

a Distributed lag model adjusting for gestational week, infant sex, asset index, self-report ethnicity, maternal age, parity, antenatal care visits, maternal BMI, and placental malaria.

*p < 0.05 **p < 0.01.

## 4 Discussion

In this exploratory study, we examined potential temporal associations between prenatal exposure to Harmattan trade winds and birth size, specifically birth weight, birth length, and head circumference among participants in the GRAPHS study in rural Ghana. Poor fetal growth is a major risk factor for perinatal mortality, whose highest rates are in sub-Saharan Africa. It is also associated with greater health risks later in life, particularly cardiovascular disease and cognitive decline (41). Studies in sub-Saharan Africa have primarily identified preventable, individual-level maternal risk factors for poor fetal growth, including nutritional status, HIV infection, and gestational hypertension (42). However, few studies have examined broader environmental risk factors that may contribute to the greater burden of poor fetal growth in this region.

Annual Harmattan trade winds in sub-Saharan West Africa bring with them air pollution from Saharan desert dust, temperature excursions, dryness and fires, and hunger stemming from crop failures and food shortages (9). Exposure to the Harmattan season is thus a bundled proxy for a suite of environmental exposures throughout pregnancy that are logistically challenging and expensive to measure continuously and collectively, let alone individually, in resource-poor settings (e.g., particulate matter from the dust winds). These exposures include elevated levels of ambient air pollution, cold temperatures, fires, and crop failure, leading to food insecurity.

We chose to examine the potential time-varying association between prenatal exposure to the Harmattan season and birth anthropometrics because there may be windows during pregnancy during which women are highly sensitive to exposure. Identifying windows of sensitivity is critical for timing public health programs and interventions that could mitigate the effects of Harmattan season exposure on poor fetal growth. These could include micronutrient supplementation to offset hunger (43) or the provision of antioxidants, as intrauterine exposure to particulate matter has been shown to promote oxidative stress and inflammation, disrupting fetal development (44, 45).

Taking this time-varying approach, we identified several weeks throughout pregnancy (gestational weeks 1–11 and 15–34) during which exposure to the Harmattan season may have been associated with reduced head circumference at birth, specifically among newborn males. Weeks 1–11 is a critical period of early fetal growth during which exposure to air pollution may disrupt placental development and the supply of nutrients and oxygen, which may impair head growth later in gestation (46). During mid- to late pregnancy (Weeks 15–34), air pollution exposure may cause inflammation, oxidative stress, and endocrine disruption, all of which could impair fetal brain and head circumference development (47). Other studies have demonstrated inverse associations between particulate matter exposure and head circumference (48, 49), including only among infant boys (50). Other Harmattan-related exposures have also been shown to be associated with lower newborn head circumference, including colder temperatures (51) and famine (52). Although the evidence is mixed (53), low head circumference at birth may be a significant risk factor for long-term adverse outcomes such as cognitive impairment, developmental delays, and chronic disease (54–56). We did not identify any potential associations between prenatal Harmattan exposure and newborn birth weight or birth length.

As with previous GRAPHS studies, sex differences in results should be interpreted with caution. Our general approach to discussing these sex-specific effects is to frame them as general sex differences in fetal growth and placental function. In this regard, male fetuses are more vulnerable to environmental exposures (perhaps for fetal and child growth outcomes), given that they grow faster from early to late gestation, whereas girls show slower growth but are more resilient to adversity. We avoid specific hypotheses about effects in a particular sex. Furthermore, the sex-specific analyses are framed as exploratory (evidence is suggestive) since we are often underpowered to detect sex differences. In this framing, sex differences need to be formally investigated in larger studies specifically designed and powered to detect them before more confident conclusions can be drawn.This exploratory study has some limitations. First, we operationalized exposure to Harmattan using a rudimentary indicator based on a general approximation of the months during which the Harmattan season occurs. We did not have access to meteorological records or more precise or sophisticated exposure metrics such as ambient particulate matter, ambient temperature, or crop yield at participants’ areas of residence. This may have resulted in non-differential misclassification of exposure, which would have biased our results toward the null or altered critical windows of gestation. Our results may have also been attenuated by the exclusion of infants born preterm from our analytic sample, as preterm birth may mediate the association between Harmattan exposure and birth size. While the DLM is a powerful tool to identify gestational windows of sensitivity, the exclusion of preterm births from our sample reflected the limitations of a traditional DLM approach in terms of its inability to handle missing data (57) or incorporate causal mediation analysis (58). Although we adjusted for several covariates, we also could not rule out potential confounding by unmeasured or unknown factors. The estimated cumulative effects of Harmattan exposure on infant head circumference may have been influenced by modeling assumptions inherent to the DLM approach, including the choice of smoothing parameters across lags. Additionally, Harmattan exposure is temporally correlated across gestational weeks, which may have limited our ability to isolate independent, lag-specific effects and influenced the magnitude of the observed cumulative estimates.

Lastly, the generalizability of our study beyond western Africa or to other dust storms is unclear given the inability to parse out individual environmental components of the Harmattan. Our study also has several strengths. We leveraged the GRAPHS cohort, which has well-characterized birth anthropometrics and ultrasound gestational age that can otherwise be difficult to capture in low-resource, rural settings. Our use of distributed lag models also enabled us to identify sensitive windows of exposure on newborn size as opposed to averaging Harmattan exposure across gestation or individual trimesters, which could miss temporal associations or bias effect estimates.

Climate change has profound implications for the Harmattan season. Studies have found that climate change is exacerbating the intensity of dust-carrying winds due to desertification and is increasing the frequency of severe cold events (9, 59, 60), both of which threaten maternal and child health. Harmattan is a natural phenomenon encompassing a variety of co-occurring issues, and studying its effects may allow us to better understand the ramifications of climate change on maternal and child health. Further research is needed on the health effects of the Harmattan season, specifically methods to more accurately define and measure environmental exposures and hazards during this time. Identifying prenatal vulnerability to Harmattan exposure may enable us to efficiently and effectively mobilize public health resources and mitigate its adverse effects on fetal growth amidst a rapidly changing global climate.

## Data Availability

The datasets presented in this article are not readily available because the data that has been used is confidential as would require reconsent of individuals and ethical approval at the country level. Requests to access the datasets should be directed to Kwaku Poku Asante, kwakupoku.asante@kintampo-hrc.org and Darby Jack, darby.jack@columbia.edu.
