# Supplementary material for: Prenatal Harmattan exposure and birth size: identifying sensitive windows in pregnancy
Source: Front Glob Womens Health. 2026 May 18;7:1576529. doi: 10.3389/fgwh.2026.1576529 (PMC13223044; doi:10.3389/fgwh.2026.1576529)
Supplement: Supplementary file 1 [file Table1.docx]

**Table S1.** Estimated Effect of Harmattan Exposure on Birth Weight by Gestational Age. Distributed lag model adjusted for infant sex, asset index, ethnicity, maternal age, parity, antenatal care visits, maternal BMI, and placental malaria. Statistically significant lags bolded.

| **Gestational week** | **Change in Birth Weight (g), 95% Confidence Interval** |
| --- | --- |
| 1 | -2.65 (-17.51, 12.20) |
| 2 | -2.53 (-16.34, 11.27) |
| 3 | -2.42 (-15.24, 10.40) |
| 4 | -2.30 (-14.22, 9.60) |
| 5 | -2.20 (-13.29, 8.88) |
| 6 | -2.11 (-12.43, 8.22) |
| 7 | -2.02 (-11.66, 7.63) |
| 8 | -1.93 (-10.97, 7.11) |
| 9 | -1.85 (-10.36, 6.66) |
| 10 | -1.78 (-9.84, 6.28) |
| 11 | -1.71 (-9.38, 5.96) |
| 12 | -1.65 (-9.00, 5.70) |
| 13 | -1.59 (-8.68, 5.50) |
| 14 | -1.54 (-8.43, 5.34) |
| 15 | -1.50 (-8.22, 5.22) |
| 16 | -1.46 (-8.06, 5.13) |
| 17 | -1.43 (-7.93, 5.07) |
| 18 | -1.40 (-7.84, 5.03) |
| 19 | -1.39 (-7.77, 5.00) |
| 20 | -1.37 (-7.73, 4.98) |
| 21 | -1.36 (-7.70, 4.97) |
| 22 | -1.36 (-7.69, 4.97) |
| 23 | -1.37 (-7.71, 4.98) |
| 24 | -1.38 (-7.75, 5.00) |
| 25 | -1.39 (-7.81, 5.03) |
| 26 | -1.41 (-7.91, 5.08) |
| 27 | -1.44 (-8.05, 5.17) |
| 28 | -1.48 (-8.24, 5.28) |
| 29 | -1.52 (-8.48, 5.45) |
| 30 | -1.56 (-8.78, 5.66) |
| 31 | -1.61 (-9.15, 5.93) |
| 32 | -1.67 (-9.60, 6.26) |
| 33 | -1.73 (-10.13, 6.66) |
| 34 | -1.80 (-10.73, 7.13) |
| 35 | -1.88 (-11.43, 7.67) |
| 36 | -1.96 (-12.20, 8.28) |
| 37 | -2.05 (-13.06, 8.96) |
| 38 | -2.14 (-14.01, 9.72) |
| 39 | -2.24 (-15.03, 10.55) |
| 40 | -2.35 (-16.14, 11.44) |

**Table S2.** Estimated Effect of Harmattan Exposure on Birth Length by Gestational Age. Distributed lag model adjusted for infant sex, asset index, ethnicity, maternal age, parity, antenatal care visits, maternal BMI, and placental malaria. Statistically significant lags bolded.

| **Gestational week** | **Change in Birth Length (cm), 95% Confidence Interval** |
| --- | --- |
| 1 | -0.02 (-0.14, 0.11) |
| 2 | -0.01 (-0.13, 0.10) |
| 3 | -0.01 (-0.12, 0.10) |
| 4 | -0.01 (-0.11, 0.09) |
| 5 | -0.01 (-0.10, 0.08) |
| 6 | -0.01 (-0.10, 0.08) |
| 7 | -0.01 (-0.09, 0.07) |
| 8 | -0.01 (-0.08, 0.07) |
| 9 | -0.01 (-0.08, 0.07) |
| 10 | -0.01 (-0.07, 0.06) |
| 11 | 0.00 (-0.07, 0.07) |
| 12 | 0.00 (-0.07, 0.06) |
| 13 | 0.00 (-0.06, 0.06) |
| 14 | 0.00 (-0.06, 0.05) |
| 15 | 0.00 (-0.06, 0.05) |
| 16 | 0.00 (-0.06, 0.05) |
| 17 | 0.00 (-0.06, 0.05) |
| 18 | 0.00 (-0.06, 0.05) |
| 19 | 0.00 (-0.06, 0.05) |
| 20 | 0.00 (-0.06, 0.05) |
| 21 | -0.01 (-0.06, 0.05) |
| 22 | -0.01 (-0.06, 0.05) |
| 23 | -0.01 (-0.06, 0.05) |
| 24 | -0.01 (-0.06, 0.05) |
| 25 | -0.01 (-0.06, 0.05) |
| 26 | -0.01 (-0.07, 0.04) |
| 27 | -0.01 (-0.07, 0.04) |
| 28 | -0.01 (-0.07, 0.04) |
| 29 | -0.01 (-0.07, 0.04) |
| 30 | -0.02 (-0.08, 0.04) |
| 31 | -0.02 (-0.08, 0.05) |
| 32 | -0.02 (-0.09, 0.05) |
| 33 | -0.02 (-0.09, 0.05) |
| 34 | -0.02 (-0.10, 0.05) |
| 35 | -0.03 (-0.11, 0.05) |
| 36 | -0.03 (-0.12, 0.06) |
| 37 | -0.03 (-0.12, 0.06) |
| 38 | -0.03 (-0.13, 0.07) |
| 39 | -0.04 (-0.15, 0.07) |
| 40 | -0.04 (-0.16, 0.08) |

**Table S3.** Estimated Effect of Harmattan Exposure on Head Circumference by Gestational Age. Distributed lag model adjusted for infant sex, asset index, ethnicity, maternal age, parity, antenatal care visits, maternal BMI, and placental malaria. Statistically significant weeks bolded.

| **Gestational week** | **Change in Head Circumference (cm), 95% Confidence Interval** |
| --- | --- |
| **1** | **-0.16 (-0.27, -0.05)** |
| **2** | **-0.14 (-0.23, -0.05)** |
| **3** | **-0.12 (-0.20, -0.04)** |
| **4** | **-0.11 (-0.08, -0.04)** |
| **5** | **-0.09 (-0.16, -0.03)** |
| **6** | **-0.08 (-0.14, -0.02)** |
| **7** | **-0.07 (-0.13, -0.02)** |
| **8** | **-0.06 (-0.12, -0.01)** |
| **9** | **-0.06 (-0.11, -0.01)** |
| **10** | **-0.05 (-0.10, 0.00)** |
| **11** | **-0.05 (-0.10, 0.00)** |
| 12 | -0.05 (-0.09, 0.00) |
| 13 | -0.04 (-0.09, 0.00) |
| 14 | -0.04 (-0.09, 0.00) |
| **15** | **-0.04 (-0.08, 0.00)** |
| **16** | **-0.04 (-0.08, 0.00)** |
| **17** | **-0.04 (-0.08, -0.01)** |
| **18** | **-0.05 (-0.08, -0.01)** |
| **19** | **-0.05 (-0.08, -0.01)** |
| **20** | **-0.05 (-0.09, -0.01)** |
| **21** | **-0.05 (-0.09, -0.02)** |
| **22** | **-0.06 (-0.09, -0.02)** |
| **23** | **-0.06 (-0.10, -0.02)** |
| **24** | **-0.06 (-0.10, -0.02)** |
| **25** | **-0.06 (-0.10, -0.02)** |
| **26** | **-0.06 (-0.11, -0.02)** |
| **27** | **-0.07 (-0.11, -0.02)** |
| **28** | **-0.07 (-0.11, -0.02)** |
| **29** | **-0.07 (-0.11, -0.02)** |
| **30** | **-0.07 (-0.11, -0.02)** |
| **31** | **-0.06 (-0.11, -0.02)** |
| **32** | **-0.06 (-0.11, -0.01)** |
| **33** | **-0.06 (-0.11, -0.01)** |
| **34** | **-0.05 (-0.10, 0.00)** |
| 35 | -0.05 (-0.10, 0.01) |
| 36 | -0.04 (-0.10, 0.02) |
| 37 | -0.03 (-0.09, 0.04) |
| 38 | -0.02 (-0.09, 0.06) |
| 39 | 0.00 (-0.09, 0.08) |
| 40 | 0.01 (-0.09, 0.11) |

**Table S4.** Estimated Effect of Harmattan Exposure on Head Circumference by Gestational Age among Males Only. Distributed lag model adjusted for asset index, ethnicity, maternal age, parity, antenatal care visits, maternal BMI, and placental malaria. Statistically significant weeks bolded.

| **Gestational week** | **Change in Head Circumference (cm), 95% Confidence Interval** |
| --- | --- |
| **1** | **-0.18 (-0.34, -0.03)** |
| **2** | **-0.16 (-0.29, -0.02)** |
| **3** | **-0.13 (-0.25, -0.02)** |
| **4** | **-0.11 (-0.22, -0.01)** |
| **5** | **-0.10 (-0.19, -0.01)** |
| 6 | -0.08 (-0.17, 0.00) |
| 7 | -0.07 (-0.15, 0.01) |
| 8 | -0.06 (-0.14, 0.01) |
| 9 | -0.05 (-0.13, 0.02) |
| 10 | -0.05 (-0.12, 0.02) |
| 11 | -0.04 (-0.11, 0.02) |
| 12 | -0.04 (-0.11, 0.02) |
| 13 | -0.04 (-0.10, 0.02) |
| 14 | -0.04 (-0.10, 0.02) |
| 15 | -0.04 (-0.10, 0.02) |
| 16 | -0.05 (-0.10, 0.01) |
| 17 | -0.05 (-0.10, 0.01) |
| 18 | -0.05 (-0.11, 0.00) |
| **19** | **-0.06 (-0.11, 0.00)** |
| **20** | **-0.06 (-0.11, -0.01)** |
| **21** | **-0.07 (-0.12, -0.01)** |
| **22** | **-0.07 (-0.12, -0.02)** |
| **23** | **-0.08 (-0.13, -0.02)** |
| **24** | **-0.08 (-0.14, -0.02)** |
| **25** | **-0.08 (-0.14, -0.02)** |
| **26** | **-0.09 (-0.15, -0.03)** |
| **27** | **-0.09 (-0.15, -0.02)** |
| **28** | **-0.09 (-0.15, -0.02)** |
| **29** | **-0.09 (-0.16, -0.02)** |
| **30** | **-0.09 (-0.16, -0.02)** |
| **31** | **-0.08 (-0.15, -0.02)** |
| **32** | **-0.08 (-0.15, -0.01)** |
| **33** | **-0.07 (-0.15, 0.00)** |
| 34 | -0.06 (-0.14, 0.01) |
| 35 | -0.05 (-0.13, 0.02) |
| 36 | -0.04 (-0.13, 0.04) |
| 37 | -0.03 (-0.12, 0.07) |
| 38 | -0.01 (-0.11, 0.10) |
| 39 | 0.01 (-0.11, 0.14) |
| 40 | 0.04 (-0.11, 0.18) |

**Table S5.** Estimated Effect of Harmattan Exposure on Head Circumference by Gestational Age among Females Only. Distributed lag model adjusted for asset index, ethnicity, maternal age, parity, antenatal care visits, maternal BMI, and placental malaria. Statistically significant weeks bolded.

| **Gestational week** | **Change in Head Circumference (cm), 95% Confidence Interval** |
| --- | --- |
| 1 | -0.12 (-0.17, 0.03) |
| 2 | -0.11 (-0.24, 0.02) |
| 3 | -0.10 (-0.21, 0.01) |
| 4 | -0.09 (-0.19, 0.01) |
| 5 | -0.09 (-0.18, 0.00) |
| 6 | -0.08 (-0.16, 0.00) |
| 7 | -0.07 (-0.15, 0.00) |
| 8 | -0.07 (-0.14, 0.00) |
| 9 | -0.06 (-0.14, 0.01) |
| 10 | -0.06 (-0.13, 0.01) |
| 11 | -0.06 (-0.12, 0.01) |
| 12 | -0.05 (-0.12, 0.01) |
| 13 | -0.05 (-0.11, 0.01) |
| 14 | -0.05 (-0.11, 0.01) |
| 15 | -0.05 (-0.11, 0.01) |
| 16 | -0.04 (-0.10, 0.01) |
| 17 | -0.04 (-0.10, 0.01) |
| 18 | -0.04 (-0.09, 0.01) |
| 19 | -0.04 (-0.09, 0.01) |
| 20 | -0.04 (-0.09, 0.01) |
| 21 | -0.04 (-0.09, 0.01) |
| 22 | -0.04 (-0.09, 0.01) |
| 23 | -0.04 (-0.09, 0.01) |
| 24 | -0.04 (-0.09, 0.02) |
| 25 | -0.04 (-0.09, 0.02) |
| 26 | -0.04 (-0.10, 0.02) |
| 27 | -0.04 (-0.10, 0.02) |
| 28 | -0.04 (-0.10, 0.03) |
| 29 | -0.04 (-0.10, 0.03) |
| 30 | -0.04 (-0.10, 0.03) |
| 31 | -0.04 (-0.10, 0.03) |
| 32 | -0.03 (-0.10, 0.03) |
| 33 | -0.03 (-0.10, 0.04) |
| 34 | -0.03 (-0.10, 0.04) |
| 35 | -0.03 (-0.11, 0.04) |
| 36 | -0.03 (-0.11, 0.05) |
| 37 | -0.03 (-0.12, 0.06) |
| 38 | -0.02 (-0.13, 0.08) |
| 39 | -0.02 (-0.14, 0.10) |
| 40 | -0.02 (-0.16, 0.12) |

**Figure S1.** Analytic Sample Flow Chart
